# Relationship Between Histological Features of Non-alcoholic Fatty Liver Disease and Ectopic Fat on Magnetic Resonance Imaging in Children and Adolescents

**DOI:** 10.3389/fped.2021.685795

**Published:** 2021-06-10

**Authors:** Eun Hye Lee, Ji Young Kim, Hye Ran Yang

**Affiliations:** ^1^Department of Pediatrics, Nowon Eulji Medical Center, Eulji University, Daejeon, South Korea; ^2^Department of Radiology, Seoul National University Bundang Hospital, Seongnam, South Korea; ^3^Department of Pediatrics, Seoul National University Bundang Hospital, Seongnam, South Korea; ^4^College of Medicine, Seoul National University, Seoul, South Korea

**Keywords:** obesity, intra-abdominal fat, biopsy, metabolic syndrome, child

## Abstract

**Objectives:** To investigate the association between ectopic fat content in the liver and pancreas, obesity-related metabolic components, and histological findings of non-alcoholic fatty liver disease (NAFLD) in children.

**Methods:** This cross-sectional study investigated 63 children with biopsy-proven NAFLD who underwent magnetic resonance imaging (MRI), anthropometry, laboratory tests, and body composition analysis. Clinical and metabolic parameters, MRI-measured hepatic fat fraction (HFF) and pancreatic fat fraction (PFF), and histological findings were analyzed.

**Results:** In a total of 63 children (48 boys, median age 12.6 years, median body mass index *z*-score 2.54), HFF was associated with histological steatosis [10.4, 23.7, and 31.1% in each steatosis grade, *P* < 0.001; Spearman's rho coefficient (rs) = 0.676; *P* < 0.001] and NAFLD activity score (rs = 0.470, *P* < 0.001), but not with lobular inflammation, hepatocyte ballooning, and hepatic fibrosis. PFF was not associated with any histological features of the liver. Waist circumference-to-height ratio and body fat percentage were associated with the steatosis grade (*P* = 0.006 and *P* = 0.004, respectively). Alanine aminotransferase was not associated with steatosis but was associated with lobular inflammation (*P* = 0.008). Lobular inflammation was also associated with high total cholesterol and low-density lipoprotein cholesterol and metabolic syndrome (*P* = 0.015, *P* = 0.036, and *P* = 0.038, respectively).

**Conclusions:** Hepatic steatosis on MRI was only associated with the histological steatosis grade, while elevated serum levels of liver enzymes and lipids were related to the severity of lobular inflammation. Therefore, MRI should be interpreted in conjunction with the anthropometric and laboratory findings in pediatric patients.

## Introduction

The prevalence of pediatric obesity has increased in recent years. Correspondingly, the incidence of non-alcoholic fatty liver disease (NAFLD) is also increasing in children since obesity is the primary etiology of NAFLD (1). NAFLD encompasses a spectrum of diseases, ranging from simple hepatic steatosis to non-alcoholic steatohepatitis (NASH), which is a progressive form of NAFLD typically associated with lobular inflammation and hepatocellular injury in addition to steatosis with or without perisinusoidal fibrosis (2, 3).

When the circulating levels of triglycerides (TG) and free fatty acid exceed the metabolic capacity of the adipose tissue, they accumulate as ectopic fat in the non-adipose tissue in patients with obesity (4, 5). Along with hepatic fat, pancreatic fat is considered an obesity-induced ectopic fat depot, which might contribute to metabolic disturbances, including diabetes mellitus (DM), hypertension (HTN), dyslipidemia, metabolic syndrome (MetS), and NAFLD (6, 7). However, little is known about the association between ectopic pancreatic fat accumulation and the histopathological severity of NAFLD, especially in children with biopsy-proven NAFLD.

According to previous studies, hepatic fibrosis at baseline is independently associated with overall mortality, liver transplantation, and liver-related events in patients with NAFLD (8, 9). However, few studies have investigated the association between the abnormalities of liver enzymes, metabolic components, and severity of histological features of NAFLD in children with biopsy-proven NAFLD.

Therefore, this study aimed to evaluate the relationship between ectopic fat measured by magnetic resonance imaging (MRI) in the liver and pancreas and the severity of each histological feature of NAFLD in children. We also aimed to investigate the relevance of histological features of the liver to anthropometry, liver enzymes, and laboratory findings of obesity-related metabolic components in pediatric patients with NAFLD.

## Materials and Methods

### Study Subjects

NAFLD is a diagnosis of exclusion based on the presence of hepatic steatosis and exclusion of non-NAFLD-related causes of hepatic steatosis (2). Liver biopsy is the current standard for defining the presence and severity of NAFLD, including the presence of NASH, and ruling out alternative and/or other concurrent chronic liver diseases, which can be challenging to exclude non-invasively (2). The optimal timing of liver biopsy to confirm the diagnosis of NAFLD and follow-up on its progression has not been established (2). Although ultrasound is widely available, a normal hepatic ultrasound cannot exclude the presence of NAFLD and therefore is not useful for diagnosis or follow-up (2). When available, MRI and magnetic resonance spectroscopy (MRS) are highly accurate for identifying steatosis (2, 10–12).

The pediatric gastroenterology, hepatology, and nutrition center in our tertiary hospital has treated many children and adolescents suspected of NAFLD referred from primary clinics, secondary hospitals, or other departments of our hospital for problems such as obesity-related comorbidities and/or abnormal laboratory results, including elevation of liver enzymes. In such cases laboratory investigations and abdominal ultrasound are performed primarily to evaluate other causes of liver disease, such as masses, gall bladder disease, and changes associated with portal hypertension (2). If the patient's clinical parameters, such as elevated liver enzymes, the severity of metabolic dysregulation, and degree of obesity, indicate evidence of ongoing disease during a follow-up period of at least 6 months or more or if there is a presence of known clinical risk factors for NASH and advanced fibrosis such as panhypopituitarism or type 2 DM, liver biopsy is considered. Since 2014, we have performed liver biopsy and abdominal MRI simultaneously.

This retrospective cross-sectional observational study included 63 children and adolescents with liver biopsy-proven NAFLD who underwent both liver biopsy and abdominal MRI performed within 2 days of each other between March 2014 and February 2020. A sample size of at least 63 was estimated to have a type I error rate of 0.05, with a power of 80% (13).

In our study, six patients with NAFLD were ≥18 years of age. One patient each was 18.1, 18.2, 18.6, 18.7, 18.8, and 19.3 years old. These six subjects were enrolled in this study despite their relatively higher age because they had been followed up continuously in the “pediatric gastroenterology, hepatology, and nutrition center” of our hospital since they were diagnosed with NAFLD at an early age. When liver biopsy and abdominal MRI were performed in these subjects, they were ≥18 years old. Moreover, in the 21st edition of the Nelson textbook of Pediatrics, the definition of adolescence includes three groups based on age: early adolescence indicates the approximate age range 10–13 years, middle adolescence is 14–17 years, and late adolescence is 18–21 years (14).

**Figure 1:**
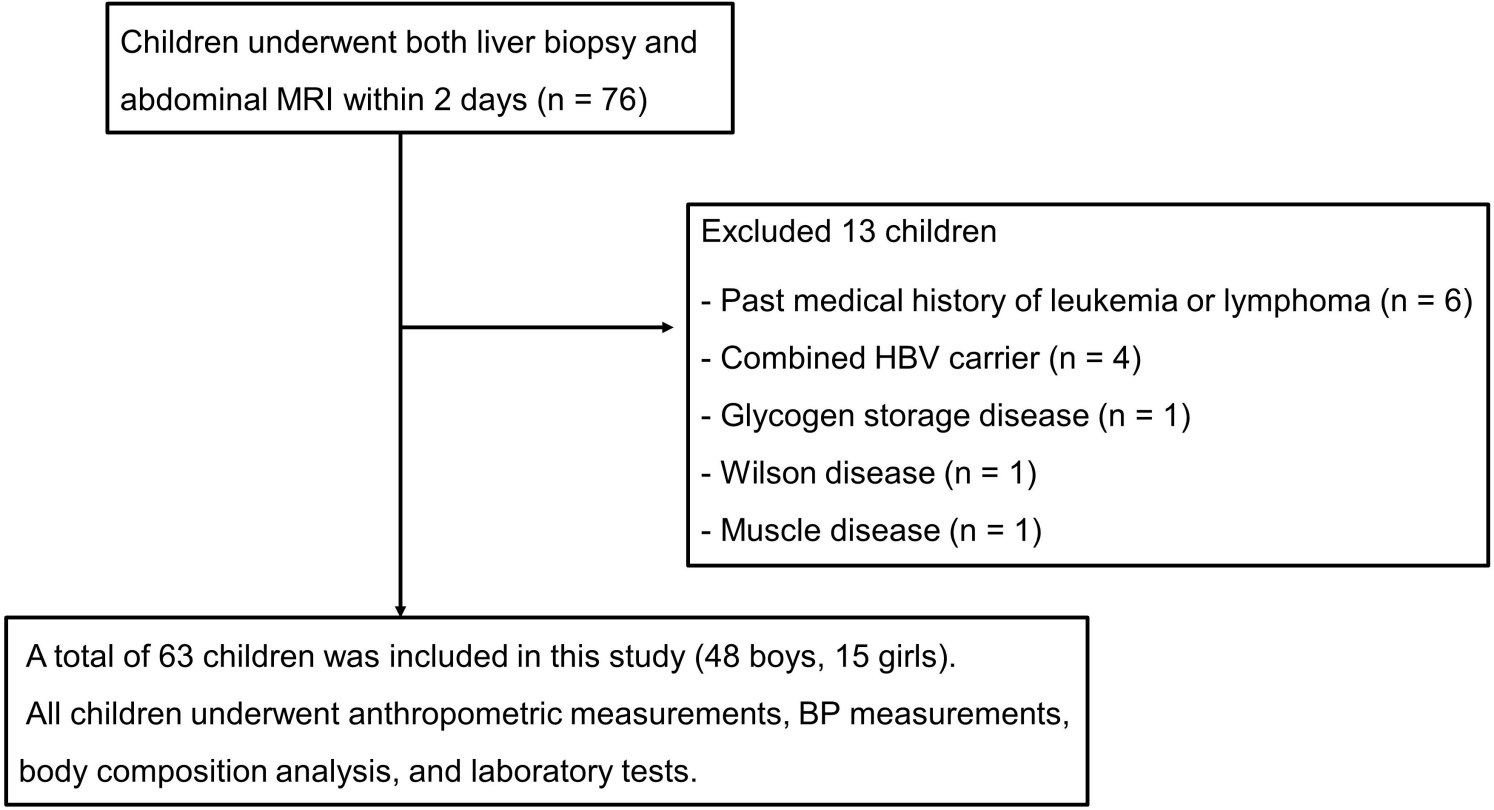
Flow diagram showing the selection of subjects. A sample size of at least 63 was estimated to have a type I error rate of 0.05, with a power of 80%. The present study included 63 children and adolescents with liver biopsy-proven non-alcoholic fatty liver disease (NAFLD) who underwent both liver biopsy and abdominal magnetic resonance imaging (MRI) performed within 2 days from March 2014 to February 2020. NAFLD was diagnosed as the presence of steatosis in ≥5% of hepatocytes in the absence of other liver diseases. Secondary etiologies of hepatic steatosis, systemic diseases, and pancreatic diseases were excluded. HBV, hepatitis B virus; BP, blood pressure.

All study subjects underwent anthropometric measurements, blood pressure (BP) measurements, body composition analysis, and laboratory tests. NAFLD was diagnosed as the presence of steatosis in ≥5% of the hepatocytes in the absence of other liver diseases. Secondary etiologies of hepatic steatosis, systemic diseases, and pancreatic diseases were excluded (Figure 1).

The study conformed to the ethical guidelines of the Declaration of Helsinki (revised in Fortaleza, Brazil, October 2013) and the recommendations of the Ethics Committee of Seoul National University Bundang Hospital. This study was approved by the Institutional Review Board of Seoul National University Bundang Hospital (IRB No. B-2103-670-104).

### Anthropometry and Blood Pressure and Body Composition Measurements

Anthropometric parameters, including height, weight, and waist circumference (WC), were measured in all children using standardized methods. Waist circumference to height ratio (WHtR), body mass index (BMI), and standard deviation score (*z*-score) were calculated. Obesity was defined as BMI ≥95th percentile, overweight as BMI between the 85th and 95th percentiles, adjusted for age and sex, according to the Korean national growth charts 2017 (15). Central obesity was defined as WC ≥90th percentile, adjusted for age and sex, according to the Korean national growth charts 2007 (16).

BP was measured during three different visits in a standardized manner (17). HTN was defined as systolic BP (SBP) or diastolic BP (DBP) ≥95th of sex-, age-, and height-specific percentiles or SBP ≥130 mm Hg or DBP ≥80 mm Hg for children aged <13 years and SBP ≥130 mm Hg or DBP ≥80 mm Hg for adolescents aged ≥13 years (17, 18). Children whose BP readings corresponded to HTN on ≥3 occasions or BP remained elevated for 1 year or more underwent 24 h ambulatory BP monitoring to confirm whether they had HTN.

Body composition was measured using bioelectrical impedance analysis (InBody J10, Biospace Co., Ltd., Seoul, Korea), and the total body fat (TBF) and fat-free mass (FFM) were recorded. The fat mass index (FMI) and fat-free mass index (FFMI) were calculated as fat mass (kg) and fat-free mass (kg), respectively, divided by the square of height (m^2^).

### Laboratory Tests

Blood samples were obtained after a 10 h overnight fast. We recorded the total cholesterol (t-chol), TG, low-density lipoprotein cholesterol (LDL-C), high-density lipoprotein cholesterol (HDL-C), aspartate aminotransferase (AST), alanine aminotransferase (ALT), total bilirubin, γ-glutamyl transferase (GGT), fasting plasma glucose (FPG), insulin, and glycated hemoglobin (HbA1c) levels.

The homeostasis model assessment of insulin resistance (HOMA-IR) was calculated using the following formula: FPG (mg/dL) × fasting insulin (μU/mL)/405 (15). The quantitative insulin sensitivity check index (QUICKI) was calculated as follows: 1/log (HOMA-IR × 405) (19).

Prediabetes and DM were defined based on the American Diabetes Association guidelines (20). Prediabetes was defined as the presence of at least one of the following: FPG 100–125 mg/dL (impaired fasting glucose), 2 h plasma glucose during oral glucose tolerance test between 140 and 199 mg/dL (impaired glucose tolerance), and an HbA1c value of 5.7–6.4% (20). DM was defined as the presence of at least one of the following: HbA1c value ≥6.5%, FPG ≥126 mg/dL, 2 h plasma glucose ≥200 mg/dL during oral glucose tolerance test, and a random plasma glucose level ≥200 mg/dL in a patient with classic symptoms of hyperglycemia (20).

Dyslipidemia was defined as the presence of at least one of the following: t-chol ≥200 mg/dL, LDL-C ≥130 mg/dL, HDL-C <40 mg/dL, TG ≥100 mg/dL (age ≤ 9 years) or ≥130 mg/dL (age ≥10 years) (21).

### Definition of Metabolic Syndrome

MetS was defined based on a revised version of the modified National Cholesterol Education Program Adult Treatment Panel III diagnostic criteria and was diagnosed in children who met at least three of the following five criteria: TG ≥110 mg/dL, HDL-C ≤ 40 mg/dL, BP ≥90th of age-, sex-, and height-specific percentiles, WC ≥90th sex-specific percentile, and FPG ≥100 mg/dL (22), not the previous criteria of FPG ≥110 mg/dL (23).

### Liver Histopathology

Liver biopsy was performed by an experienced pediatric radiologist under ultrasound guidance, and the findings were interpreted by an expert liver pathologist who was blinded to the patients' clinical data.

All biopsy specimens were evaluated based on the NAFLD Clinical Research Network criteria (24), and the NAFLD activity score (NAS) was assessed (25). The scores for degree of steatosis, lobular inflammation, portal inflammation, and hepatocyte ballooning degeneration were calculated (steatosis 0–3, lobular inflammation 0–3, portal inflammation 0–2, hepatocyte ballooning 0–2) (24). Hepatic fibrosis was categorized into stages 0–4 (stage 0, no fibrosis; stage 1, perisinusoidal or periportal fibrosis; stage 2, perisinusoidal and portal/periportal fibrosis; stage 3, bridging fibrosis; stage 4, cirrhosis) (24). NAS was calculated using an eight-point scale as the sum of the scores for steatosis, lobular inflammation, and hepatocyte ballooning (25). The subjects were also divided into the following NAS subgroups: NAS 0–2, NAS 3–4, and NAS ≥5.

### Magnetic Resonance Imaging-Based Measurement of Hepatic and Pancreatic Fat Fractions

Abdominal MRI examinations were performed using a 3.0 T MR scanner (Ingenia, Philips Healthcare, Best, Netherlands). The modified DIXON-Quant sequence was obtained during a single breath hold, which automatically reconstructed a proton density fat fraction (PDFF) map. We obtained the maps of water, fat, fat fraction, R2^*^, and T2^*^ by post-processing the acquired images using the software provided by the manufacturer. All data were transferred to the IntelliSpace Portal software (version 10.0; Philips, Amsterdam, The Netherlands). Selection of the region of interest and fat fraction measurements were performed by an expert pediatric radiologist who was blinded to the patients' clinical and histopathological data. Hepatic fat fraction (HFF) measurements were performed by drawing two different regions of interest in the right and left hepatic lobes. MRI-PDFF is known to have an excellent diagnostic value for the assessment of hepatic fat content in patients with NAFLD (26). HFF ≥5.0% on MRI-PDFF is defined as a fatty liver because MRI-PDFF is highly accurate compared to the histological diagnosis of steatosis (26). Regions of interest for pancreatic fat fraction (PFF) measurements were selected in the head, body, and tail of the pancreas. The normal range of PFF to define fatty pancreas has not yet been established.

### Statistical Analysis

Descriptive characteristics are presented as mean ± SD for normally distributed variables or as medians and ranges for non-normally distributed variables. Categorical measurements are expressed as absolute numbers and percentages.

Intergroup differences were evaluated using ANOVA or an independent *t*-test for parametric variables and the Kruskal-Wallis test or the Mann-Whitney *U*-test for non-parametric variables. Categorical variables were compared using the *chi*-square or Fisher's exact test. Correlations between the continuous and/or ordinal variables were tested using the Pearson's correlation matrix or the Spearman rank correlation matrix according to the property of variables.

A two-sided *P*-value of <0.05 was considered statistically significant. All statistical analyses were performed using PASW Statistics software (version 25.0; SPSS Inc., Chicago, IL, USA).

## Results

### Patient Characteristics

We investigated 63 children with biopsy-proven NAFLD (48 boys, 15 girls; median age 12.6 years, ranging 5.6–19.3 years). The MRI-measured ectopic fat content and histological findings of the liver are shown in Table 1. Most subjects were overweight or obese (median BMI *z*-score 2.54, range −1.10–5.10). Thirteen (20.6%) children were overweight, 45 (71.4%) children were obese, and only 5 (7.9%) children were within the normal range (BMI <85th percentile) (Table 2). Central obesity was confirmed in 54 (85.7%) children. Elevated BP was noted in 9 (14.3%) children, and HTN was diagnosed in 16 (25.4%) children. Prediabetes was diagnosed in 14 (22.2%), and DM in 9 (14.3%) children. Dyslipidemia was observed in 42 (66.7%) and MetS was diagnosed in 25 (39.7%) children (Table 2).

**Table 1:** Magnetic resonance imaging based-fat fractions and histopathologic features of the liver in 63 children with non-alcoholic fatty liver disease.

**Variable**	**Data**
Magnetic resonance imaging-based fat fraction	
Hepatic fat fraction (%)	24.3 (4.7–49.9)
Pancreatic fat fraction (%)	3.8 (0.4–26.9)
Liver histopathology	
Steatosis grade (*n*, %)	
0	0
1	13 (20.6%)
2	18 (28.6%)
3	32 (50.8%)
Lobular inflammation grade (*n*, %)	
0	0
1	32 (50.8%)
2	29 (46.0%)
3	2 (3.2%)
Portal inflammation grade (*n*, %)	
0	24 (38.1%)
1	38 (60.3%)
2	1 (1.6%)
Ballooning degeneration grade (*n*, %)	
0	33 (52.4%)
1	24 (38.1%)
2	6 (9.5%)
Fibrosis stage (*n*, %)	
0	11 (17.5%)
1	41 (65.1%)
2	11 (17.5%)
3	0
4	0
NAFLD activity score (*n*, %)	
0–2	8 (12.7%)
3–4	24 (38.1%)
5–8	31 (49.2%)
NASH classification (*n*, %)	
NAFLD, non-NASH	19 (30.2%)
NASH	44 (69.8%)

*Values are presented as median (range) or numbers (%). NAFLD, non-alcoholic fatty liver disease; NASH, non-alcoholic steatohepatitis*.

**Table 2:** Comparison of demographic, anthropometric, laboratory, and magnetic resonance imaging-measured fat fraction findings of 63 children with non-alcoholic fatty liver disease according to liver histologic steatosis grade.

**Variable**	**Steatosis** **Grade 1** **(*n* = 13)**	**Steatosis** **Grade 2** **(*n* = 18)**	**Steatosis** **Grade 3** **(*n* = 32)**	**Total patients** **(*n* = 63)**	***P*-value**
Sex (boys: girls)	9 (69.2%): 4 (30.8%)	14 (77.8%): 4 (22.2%)	25 (78.1%): 7 (21.9%)	48 (76.2%): 15 (23.8%)	0.857^*^
Age, yr	11.2 (5.6–19.3)	12.8 (10.5–18.8)	12.0 (6.1–18.6)	12.6 (5.6–19.3)	0.165
Height (cm)	155.8 (122.4–184.3)	158.1 (141.9–179.9)	150.8 (123.8–184.7)	155.8 (122.4–184.7)	0.663
Height *z*-score	1.15 (−2.90–2.82)	0.27 (−3.40–1.25)	0.88 (−1.01–2.17)	0.87 (−3.40–2.82)	0.082
Weight (kg)	63.3 (37.9–110.7)	73.4 (39.2–110.6)	63.6 (36.4–30.9)	65.3 (36.4–130.9)	0.469
Weight *z*-score	1.95 (−0.37–3.75)	1.89 (−2.80–3.68)	2.38 (0.41–5.01)	2.16 (−2.80–5.01)	0.160
WC (cm)	87.5 (75.0–105.7)	90.5 (67.0–113.4)	93.0 (75.0–120.0)	89.1 (67.0–120.0)	0.247
WHtR	0.55 (0.50–0.69)	0.57 (0.46–0.69)	0.60 (0.52–0.69)	0.59 (0.46–0.69)	0.026
BMI	25.3 (20.0–32.6)	27.9 (18.6–35.2)	27.5 (21.6–38.4)	26.7 (18.6–38.4)	0.198
BMI *z*-score	1.78 (0–4.56)	2.22 (−1.10–4.19)	2.65 (0.89–5.10)	2.54 (−1.10–5.10)	0.122
Fat mass (kg)	20.0 (12.9–39.7)	24.5 (8.2–39.7)	25.4 (12.6–54.0)	23.2 (8.2–54.0)	0.210
Fat mass (%)	32.9 (25.6–43.8)	34.7 (22.3–48.2)	39.4 (24.1–67.2)	35.8 (22.3–67.2)	0.017
FFM (kg)	41.5 (27.7–71.0)	45.5 (28.6–74.2)	34.5 (23.5–79.7)	41.0 (23.5–79.7)	0.695
FFM (%)	67.1 (56.3–74.4)	65.3 (51.8–77.7)	60.6 (32.8–75.8)	64.1 (32.8–77.7)	0.019
FMI	8.2 (5.8–12.7)	8.8 (3.9–17.1)	10.4 (7.1–18.8)	9.9 (3.9–18.8)	0.018
FFMI	17.3 (13.2–20.9)	18.5 (13.6–22.9)	15.8 (9.2–23.4)	17.1 (9.2–23.4)	0.778
mSBP (mmHg)	114.0 (100–144)	121 (100–148)	117.0 (106–159)	118.0 (100–159)	0.271
mDBP (mmHg)	62.0 (52–75)	67.0 (52–84)	63.5 (53–90)	63.0 (52–90)	0.285
AST (IU/L)	49.0 (17–161)	52.0 (27–196)	61.0 (23–226)	54.0 (17–226)	0.429
ALT (IU/L)	88.0 (21–331)	120.5 (20–287)	134.5 (48–366)	122.0 (20–366)	0.301
AST/ALT	0.53 (0.40–1.26)	0.51 (0.31–1.52)	0.47 (0.28–1.26)	0.48 (0.28–1.52)	0.133
t-bil (mg/dL)	0.6 (0.3–0.9)	0.7 (0.2–1.3)	0.5 (0.3–1.1)	0.6 (0.2–1.3)	0.991
GGT (IU/L)	28.0 (12–117)	36.5 (19–133)	43.0 (17–184)	38.0 (12–184)	0.186
t-chol (mg/dL)	172.0 (105–277)	176.0 (122–258)	187.5 (122–275)	180.0 (105–277)	0.458
TG (mg/dL)	130.0 (56–364)	99.0 (48–357)	122.5 (54–280)	121.0 (48–364)	0.851
HDL-C (mg/dL)	45.0 (28–68)	46.0 (37–67)	45.5 (28–61)	45.0 (28–68)	0.948
LDL-C (mg/dL)	96.0 (55–168)	105.5 (66–146)	110.5 (61–175)	108.0 (55–175)	0.412
FPG (mg/dL)	90.0 (71–113)	102.5 (76–270)	90.5 (71–179)	93.0 (71–270)	0.005
Insulin (mIU/L)	19.9 (8.9–75.6)	21.7 (11.1–47.9)	20.9 (4.6–75.4)	20.9 (4.6–75.6)	0.894
HbA1c (%)	5.5 (5.0–7.3)	5.9 (5.0–11.0)	5.3 (5.1–13.5)	5.4 (5.0–13.5)	0.248
HOMA-IR	4.2 (2.1–17.0)	6.0 (2.1–14.0)	4.7 (1.0–14.5)	5.1 (1.0–17.0)	0.295
QUICKI	0.31 (0.26–0.34)	0.30 (0.27–0.34)	0.31 (0.27–0.39)	0.30 (0.26–0.39)	0.295
MRI HFF (%)	10.4 (4.7–21.0)	23.7 (9.2–33.0)	31.1 (11.6–49.9)	24.3 (4.7–49.9)	<0.001
MRI PFF (%)	2.7 (1.2–11.9)	4.4 (0.4–26.9)	3.6 (1.4–15.8)	3.8 (0.4–26.9)	0.241
BMI: Normal/Overweight/Obesity (*n*, %)	1 (7.7%)/5 (38.5%)/7 (53.8%)	3 (16.7%)/5 (27.8%)/10 (55.6%)	1 (3.1%)/3 (9.4%)/28 (87.5%)	5 (7.9%)/13 (20.6%)/45 (71.4%)	0.032^*^ 0.029^†^
Central obesity (*n*, %)	11 (84.6%)	13 (72.2%)	30 (93.8%)	54 (85.7%)	0.115^*^
BP category: Normal/EBP + HTN (*n*, %)	9 (69.2%)/4 (30.8%)	9 (50%)/9 (50%)	20 (62.5%)/12 (37.5%)	38 (60.3%)/25 (39.7%)	0.523^‡^
Dyslipidemia (*n*, %)	7 (53.8%)	12 (66.7%)	23 (71.9%)	42 (66.7%)	0.509^‡^
DM category: Normal/PreDM + DM (*n*, %)	9 (69.2%)/4 (30.8%)	6 (33.3%)/12 (66.7%)	25 (78.1%)/7 (21.9%)	40 (63.5%)/23 (36.5%)	0.006^‡^
MetS (*n*, %)	3 (23.1%)	8 (44.4%)	14 (43.8%)	25 (39.7%)	0.389^‡^

*P-value was calculated by Kruskal-Wallis test*.

* *P-value was calculated by Fisher's exact test*.

† *P-value was calculated for linear trend of obesity proportion*.

‡ *P-value was calculated by Chi-square test. Values are presented as median (range) or numbers (%)*.

*WC, waist circumference; WHtR, waist circumference-to-height ratio; BMI, body mass index; FFM, fat-free mass; FMI, fat mass index = fat mass(kg)/height(m)^2^; FFMI, fat-free mass index = FFM(kg)/height(m)^2^; mSBP, mean systolic blood pressure; mDBP, mean diastolic blood pressure; AST, aspartate aminotransferase; ALT, alanine aminotransferase; t-bil, total bilirubin; GGT, γ-glutamyl transferase; t-chol, total cholesterol; HDL-C, high density lipoprotein cholesterol; LDL-C, low density lipoprotein cholesterol; FPG, fasting plasma glucose; HbA1c, glycated hemoglobin; HOMA-IR, homeostatic model assessment of insulin resistance; QUICKI, Quantitative insulin sensitivity check index; MRI, magnetic resonance imaging; HFF, hepatic fat fraction; PFF, pancreatic fat fraction; BP, blood pressure; EBP, elevated BP; HTN, hypertension; DM, diabetes mellitus; PreDM, prediabetes; MetS, metabolic syndrome*.

### Comparison Between MRI PDFF-Measured HFF and PFF According to Histological Features of NAFLD

**Figure 2:**
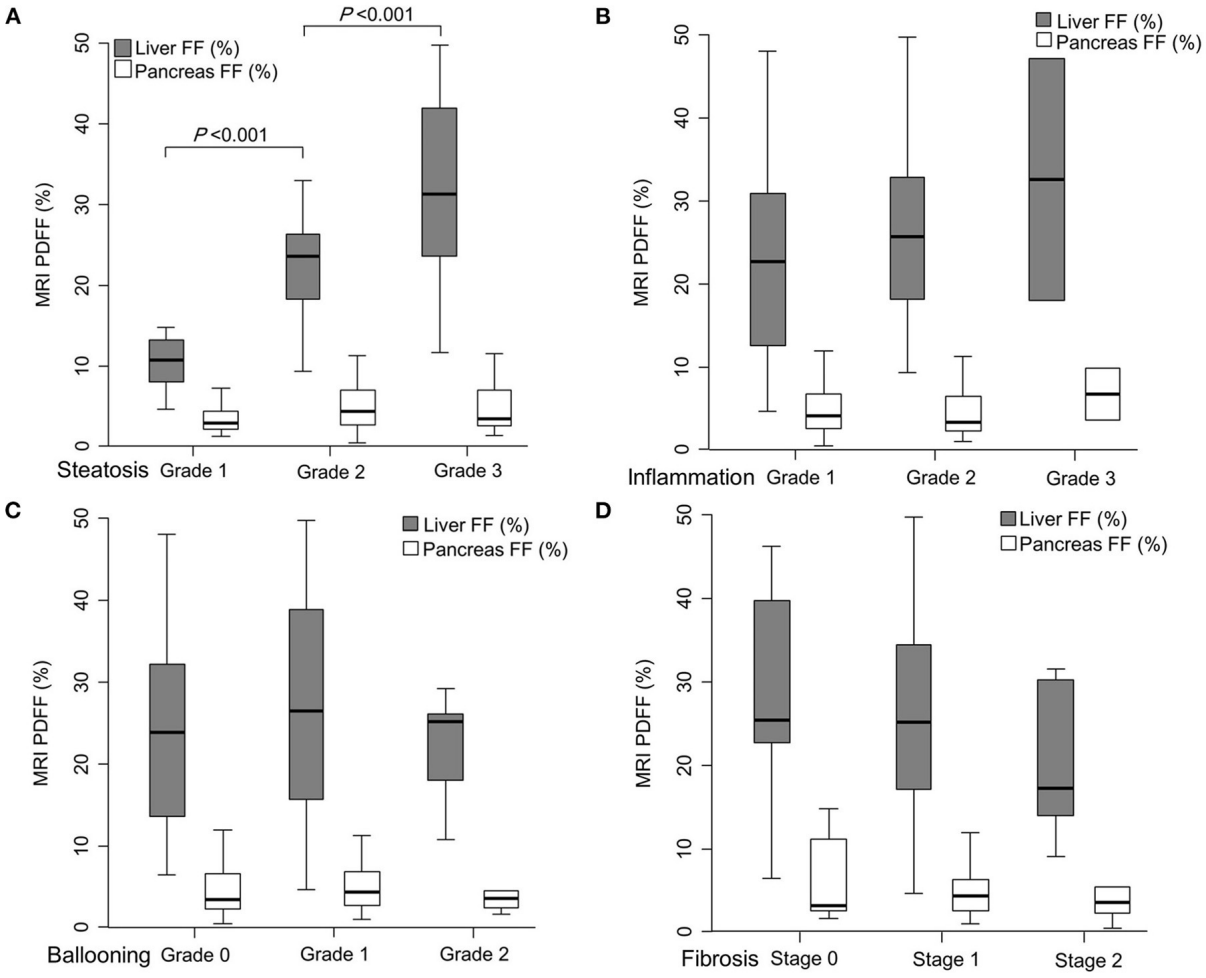
Comparison of magnetic resonance imaging (MRI) proton density fat fraction (PDFF)-measured hepatic fat fraction (HFF) and pancreatic fat fraction (PFF) according to each histological feature of non-alcoholic fatty liver disease (NAFLD); steatosis grade **(A)**, lobular inflammation grade **(B)**, ballooning degeneration grade **(C)**, and hepatic fibrosis stage **(D)**. HFF significantly increased with the rise of hepatic steatosis grade (10.4, 23.7, and 31.1% in each steatosis grade, *P* < 0.001). However, HFF was not significantly different when observing other histological features of NAFLD, such as lobular inflammation, ballooning degeneration, and hepatic fibrosis. PFF was not related to any liver histologic features of NAFLD.

When HFF and PFF were compared according to the severity of each histological feature, HFF significantly increased with increase in the grade of hepatic steatosis (10.4, 23.7, and 31.1% in steatosis grade 1, 2, and 3, respectively, *P* < 0.001) (Figure 2; Table 2). HFF correlated with the grade of steatosis [Spearman's rho coefficient (rs) = 0.676, *P* < 0.001]. However, HFF was not significantly different with different grades of other histological features of NAFLD, such as lobular inflammation, ballooning degeneration, and fibrosis (Figure 2; Tables 3–5). Furthermore, HFF did not correlate with other histological features of NAFLD, except for hepatic steatosis.

**Table 3:** Comparison of demographic, anthropometric, laboratory, and magnetic resonance imaging-measured fat fraction findings of 63 children with non-alcoholic fatty liver disease by liver histologic lobular inflammation grade.

**Variable**	**Lobular inflammation Grade 1** **(*n* = 32)**	**Lobular inflammation Grade 2** **(*n* = 29)**	**Lobular inflammation Grade 3** **(*n* = 2)**	***P*-value**
Sex (boys: girls)	25 (78.1%): 7 (21.9%)	22 (75.9%): 7 (24.1%)	1 (50.0%): 1 (50.0%)	0.638^*^
Age, yr	12.3 (5.6–19.3)	12.7 (7.5–18.8)	13.1 (10.3–15.9)	0.708
Height (cm)	152.7 (122.4–184.5)	158.2 (134.5–184.7)	153.0 (142.5–163.4)	0.692
Height *z*-score	0.94 (−3.40–2.82)	0.86 (−1.78–2.17)	0.51 (0.37–0.65)	0.631
Weight (kg)	63.2 (36.4–117.5)	73.9 (41.2–130.9)	69.1 (58.3–79.9)	0.239
Weight *z*-score	2.01 (−2.80–4.19)	2.34 (0.69–5.01)	2.60 (2.37–2.82)	0.531
WC (cm)	87.3 (67.0–111.5)	93.0 (75.0–120.0)	93.5 (93.0–94.0)	0.141
WHtR	0.57 (0.46–0.69)	0.61 (0.51–0.69)	0.62 (0.57–0.66)	0.139
BMI	25.4 (18.6–34.5)	28.6 (21.6–38.4)	29.3 (28.7–29.9)	0.064
BMI *z*-score	2.27 (−1.10–4.56)	2.69 (0.68–5.10)	3.03 (3.0–3.05)	0.156
Fat mass (kg)	21.5 (8.2–54.0)	26.5 (13.5–52.4)	29.1 (25.8–32.3)	0.103
Fat mass (%)	35.0 (22.3–67.2)	36.5 (25.6–48.2)	43.1 (39.3–46.8)	0.101
FFM (kg)	40.0 (23.5–79.7)	42.6 (27.2–76.3)	39.6 (29.3–49.8)	0.678
FFM (%)	65.0 (32.8–77.7)	63.5 (51.8–74.4)	56.9 (53.2–60.7)	0.098
FMI	8.8 (3.9–18.8)	11.0 (6.2–17.1)	12.4 (12.1–12.7)	0.042
FFMI	16.9 (9.2–12.4)	18.0 (13.2–22.9)	16.5 (14.4–18.7)	0.492
mSBP (mmHg)	116.5 (100–150)	120.0 (106–159)	120.0 (114–126)	0.331
mDBP (mmHg)	63.0 (52–75)	66.0 (52–90)	60.5 (60–61)	0.234
AST (IU/L)	47.5 (17–161)	61.0 (30–226)	184.0 (178–190)	0.004
ALT (IU/L)	93.5 (20–331)	139.0 (31–342)	337.0 (308–366)	0.010
AST/ALT	0.47 (0.28–1.45)	0.49 (0.31–1.52)	0.55 (0.49–0.62)	0.695
Total bilirubin (mg/dL)	0.6 (0.3–0.9)	0.6 (0.2–1.3)	0.6 (0.5–0.6)	0.789
GGT (IU/L)	33.0 (12–158)	40.0 (19–184)	89.5 (71–108)	0.103
Total cholesterol (mg/dL)	170.5 (105–277)	197.0 (122–258)	246.5 (244–249)	0.021
Triglyceride (mg/dL)	114.0 (48–364)	125.0 (54–293)	180 (118–242)	0.637
HDL-C (mg/dL)	45.0 (28–58)	46.0 (28–68)	49.0 (45–53)	0.717
LDL-C (mg/dL)	97.5 (55–175)	117.0 (61–164)	152.0 (150–154)	0.036
FPG (mg/dL)	92.0 (71–179)	95.0 (71–270)	126.5 (105–148)	0.189
Insulin (mIU/L)	20.5 (4.6–75.6)	22.4 (9.1–75.4)	17.5 (6.4–28.5)	0.661
HbA1c (%)	5.3 (5.0–13.5)	5.4 (5.1–11.0)	8.4 (6.3–10.5)	0.060
HOMA-IR	4.7 (1.0–17.0)	5.9 (2.0–14.5)	4.9 (2.3–7.4)	0.719
QUICKI	0.31 (0.26–0.39)	0.30 (0.27–0.34)	0.31 (0.29–0.34)	0.719
MRI HFF (%)	23.0 (4.7–48.0)	25.7 (9.2–49.9)	32.6 (18.0–47.2)	0.346
MRI PFF (%)	4.1 (0.4–14.8)	3.3 (1.0–26.9)	6.7 (3.6–9.9)	0.668
BMI: Normal/Overweight/Obesity (*n*, %)	4 (12.5%)/7 (21.9%)/21 (65.6%)	1 (3.4%)/6 (20.7%)/22 (75.9%)	0 (0%)/0 (0%)/2 (100%)	0.670^*^
Central obesity (*n*, %)	26 (81.3%)	26 (89.7%)	2 (100%)	0.617^*^
Normal BP/EBP + HTN (*n*, %)	23 (71.9%)/9 (28.1%)	14 (48.3%)/15 (51.7%)	1 (50.0%)/1 (50.0%)	0.088^*^
Dyslipidemia (*n*, %)	18 (56.3%)	22 (75.9%)	2 (100%)	0.231^*^
Normal/PreDM + DM (*n*, %)	23 (71.9%)/9 (28.1%)	17 (58.6%)/12 (41.4%)	0 (0%)/2 (100%)	0.078^*^
MetS (*n*, %)	9 (28.1%)	14 (48.3%)	2 (100%)	0.050^*^ 0.038^†^

*P-value was calculated by Kruskal-Wallis test*.

* *P-value was analyzed by Fisher's exact test*.

† *P-value was calculated for linear trend of metabolic syndrome proportion. Values are presented as median (range) or numbers (%)*.

*WC, waist circumference; WHtR, waist circumference-to-height ratio; BMI, body mass index; FFM, fat free mass; FMI, fat mass index = fat mass(kg)/height(m)^2^; FFMI, fat free mass index = FFM(kg)/height(m)^2^; mSBP, mean systolic blood pressure; mDBP, mean diastolic blood pressure; AST, aspartate aminotransferase; ALT, alanine aminotransferase; GGT, γ-glutamyl transferase; HDL-C, high density lipoprotein cholesterol; LDL-C, low density lipoprotein cholesterol; FPG, fasting plasma glucose; HbA1c, glycated hemoglobin; HOMA-IR, homeostatic model assessment of insulin resistance; QUICKI, Quantitative insulin sensitivity check index; MRI, magnetic resonance imaging; HFF, hepatic fat fraction; PFF, pancreatic fat fraction; BP, blood pressure; EBP, elevated BP; HTN, hypertension; DM, diabetes mellitus; PreDM, prediabetes; MetS, metabolic syndrome*.

HFF was significantly different among the three groups divided by NAS (10.1% in NAS 0–2, 24.3% in NAS 3–4, and 27.0% in NAS ≥5, *P* = 0.002). HFF correlated with NAS (rs = 0.470, *P* < 0.001).

However, PFF did not significantly differ according to the histological features of NAFLD (Tables 2–5). PFF was also not correlated with any histological characteristic of NAFLD. Furthermore, PFF was not correlated with HFF (Pearson's correlation coefficient = 0.170, *P* = 0.191).

### Association Between Liver Histological Features and Liver Enzymes

Liver enzymes, such as AST, ALT, and GGT were not significantly different among the subgroups based on the grades of hepatic steatosis, ballooning degeneration, portal inflammation, and stage of hepatic fibrosis (Tables 2, 4–6).

**Table 4:** Comparison of demographic, anthropometric, laboratory, and magnetic resonance imaging-measured fat fraction findings of 63 children with non-alcoholic fatty liver disease by liver histologic ballooning degeneration grade.

**Variable**	**Hepatocyte ballooning** **Grade 1** **(*n* = 33)**	**Hepatocyte ballooning** **Grade 2** **(*n* = 24)**	**Hepatocyte ballooning** **Grade 3** **(*n* = 6)**	***P*-value**
Sex (boys: girls)	24 (72.7%): 9 (27.3%)	21 (87.5%): 3 (12.5%)	3 (50.0%): 3 (50.0%)	0.099^*^
Age, yr	12.4 (5.6–19.3)	12.4 (8.7–17.8)	14.7 (7.7–16.5)	0.882
Height (cm)	153.7(122.4–184.5)	157.1 (134.5–184.7)	156.1 (136.2–168.9)	0.759
Height *z*-score	0.98 (−3.40–2.17)	0.75 (−1.38–2.82)	0.50 (−1.78–1.93)	0.522
Weight (kg)	64.6 (36.4–117.5)	67.3 (46.3–130.9)	67.9 (43.6–79.9)	0.481
Weight *z*-score	2.40 (−2.80–4.19)	2.00 (0.99–5.01)	1.71 (0.69–3.0)	0.621
WC (cm)	87.0 (67.0–113.0)	92.0 (79.0–120.0)	89.5 (75.0–93.5)	0.341
WHtR	0.59 (0.46–0.69)	0.62 (0.51–0.69)	0.58 (0.52–0.65)	0.257
BMI	26.7 (18.6–34.5)	27.4 (21.9–38.4)	26.5 (21.6–31.0)	0.501
BMI *z*-score	2.46 (−1.10–4.56)	2.60 (0.68–5.10)	2.49 (1.34–3.23)	0.764
Fat mass (kg)	22.7 (8.2–54.0)	23.7 (15.9–52.4)	25.4 (13.8–32.3)	0.317
Fat mass (%)	34.8 (22.3–67.2)	40.4 (26.3–48.2)	36.6 (31.4–45.1)	0.100
FFM (kg)	22.7 (8.2–54.0)	23.7 (15.9–52.4)	25.4 (13.8–32.3)	0.317
FFM (%)	65.3 (32.8–77.7)	59.5 (51.8–73.7)	63.4 (54.8–68.7)	0.101
FMI	8.9 (3.9–18.8)	10.5 (6.2–17.1)	10.5 (6.8–12.1)	0.196
FFMI	16.6 (9.2–23.4)	17.5 (14.4–22.6)	17.9 (13.2–20.0)	0.914
mSBP (mmHg)	118.0 (100–150)	118.5 (102–159)	117.0 (111–133)	0.882
mDBP (mmHg)	63.0 (52–81)	64.0 (52–90)	63.5 (55–75)	0.371
AST (IU/L)	51.0 (17–196)	59.5 (21–216)	88.0 (25–226)	0.334
ALT (IU/L)	115.0 (20–287)	125.0 (21–342)	183.0 (48–366)	0.247
AST/ALT	0.47 (0.29–1.45)	0.49 (0.28–1.52)	0.52 (0.42–0.77)	0.755
Total bilirubin (mg/dL)	0.6 (0.3–1.3)	0.6 (0.2–1.1)	0.5 (0.3–1.1)	0.922
GGT (IU/L)	35.0 (12–158)	44.0 (12–117)	34.0 (20–184)	0.657
Total cholesterol (mg/dL)	169.0 (105–277)	198.0 (120–275)	192.5 (175–244)	0.082
Triglyceride (mg/dL)	130.0 (48–364)	98.5 (64–280)	101.5 (64–144)	0.278
HDL-C (mg/dL)	46.0 (36–68)	45.0 (28–61)	48.5 (37–53)	0.861
LDL-C (mg/dL)	99.0 (55–168)	119.5 (61–175)	119.5 (99–150)	0.060
FPG (mg/dL)	91.0 (76–189)	95.5 (71–270)	98.5 (77–148)	0.954
Insulin (mIU/L)	22.0 (4.6–75.6)	22.6 (9.5–75.4)	17.2 (6.4–19.3)	0.097
HbA1c (%)	5.5 (5.0–8.0)	5.3 (5.1–13.5)	5.3 (5.1–10.5)	0.826
HOMA-IR	4.8 (1.0–17.0)	5.9 (2.1–14.5)	3.4 (2.3–4.7)	0.085
QUICKI	0.30 (0.26–0.39)	0.30 (0.27–0.34)	0.32 (0.31–0.34)	0.085
MRI HFF (%)	23.9 (6.4–48.1)	26.5 (4.7–49.9)	25.2 (10.7–29.2)	0.734
MRI PFF (%)	3.4 (0.4–26.9)	4.4 (1.0–15.8)	3.6 (1.7–10.4)	0.644
BMI: Normal/Overweight/Obesity (*n*, %)	4 (12.1%)/6 (18.2%)/23 (69.7%)	1 (4.2%)/5 (20.8%)/18 (75.0%)	0 (0%)/2 (33.3%)/4 (66.7%)	0.763^*^
Central obesity (*n*, %)	26 (78.8%)	23 (95.8%)	5 (83.3%)	0.145^*^
Normal BP/EBP + HTN (*n*, %)	21 (63.6%)/12 (36.4%)	14 (58.3%)/10 (41.7%)	3 (50.0%)/3 (50.0%)	0.804^*^
Dyslipidemia (*n*, %)	23 (69.7%)	16 (66.7%)	3 (50.0%)	0.679^*^
Normal/PreDM + DM (*n*, %)	20 (60.6%)/13 (39.4%)	15 (62.5%)/9 (37.5%)	5 (83.3%)/1 (16.7%)	0.689^*^
MetS (*n*, %)	13 (39.4%)	9 (37.5%)	3 (50.0%)	0.564^*^

*P-value was calculated by Kruskal-Wallis test*.

* *P-value was analyzed by Fisher's exact test. Values are presented as median (range) or numbers (%)*.

*WC, waist circumference; WHtR, waist circumference-to-height ratio; BMI, body mass index; FFM, fat free mass; FMI, fat mass index = fat mass(kg)/height(m)^2^; FFMI, fat free mass index = FFM(kg)/height(m)^2^; mSBP, mean systolic blood pressure; mDBP, mean diastolic blood pressure; AST, aspartate aminotransferase; ALT, alanine aminotransferase; GGT, γ-glutamyl transferase; HDL-C, high density lipoprotein cholesterol; LDL-C, low density lipoprotein cholesterol; FPG, fasting plasma glucose; HbA1c, glycated hemoglobin; HOMA-IR, homeostatic model assessment of insulin resistance; QUICKI, Quantitative insulin sensitivity check index; MRI, magnetic resonance imaging; HFF, hepatic fat fraction; PFF, pancreatic fat fraction; BP, blood pressure; EBP, elevated BP; HTN, hypertension; DM, diabetes mellitus; PreDM, prediabetes; MetS, metabolic syndrome*.

However, AST and ALT significantly increased according to the grade of lobular inflammation (47.5 in grade 1, 61.0 in grade 2, and 184.0 IU/L in grade 3, respectively, for AST, *P* = 0.004; 93.5, 139.0, and 337.0 IU/L, respectively, for ALT, *P* = 0.010) (Table 3). AST and ALT levels were significantly correlated with lobular inflammation (rs = 0.398, *P* = 0.001, and rs = 0.333, *P* = 0.008, respectively).

AST and ALT were also correlated with NAS (rs = 0.302, *P* = 0.016, and rs = 0.303, *P* = 0.016, respectively).

### Comparison Between Histological Features of the Liver and Measurements of Body Composition

As the grade of hepatic steatosis increased, the TBF percentage increased significantly (32.9% in grade 1, 34.7% in grade 2, and 39.4% in grade 3, *P* = 0.017), and correspondingly, the FFM percentage significantly decreased (67.1, 65.3, and 60.6%, respectively, *P* = 0.019) (Table 2). TBF percentage was positively correlated with the grade of steatosis (rs = 0.363, *P* = 0.004), and the FFM percentage was negatively correlated with the grade of steatosis (rs = −0.359, *P* = 0.005). Likewise, FMI was significantly increased with the grade of hepatic steatosis (8.2 in grade 1, 8.8 in grade 2, and 10.4 in grade 3, *P* = 0.018) (Table 2) and FMI was positively correlated with the grade of steatosis (rs = 0.366, *P* = 0.004).

Furthermore, FMI significantly increased according to the grade of lobular inflammation (8.8 in grade 1, 11.0 in grade 2, and 12.4 in grade 3, *P* = 0.042), and FMI was positively correlated with the grade of lobular inflammation (rs = 0.309, *P* = 0.016).

### Association Between Liver Histological Features and Metabolic Components

As the grade of hepatic steatosis increased, the proportion of obesity significantly increased (53.8% in grade 1, 55.6% in grade 2, and 87.5% in grade 3; *P* = 0.032, *P* for linear trend = 0.029) (Table 2). WHtR also significantly increased as the grade of steatosis increased (0.55, 0.57, and 0.60, respectively, *P* = 0.026) (Table 2), and it was correlated with the steatosis grade (rs = 0.342, *P* = 0.006).

As the grade of lobular inflammation increased, t-chol and LDL-C significantly increased (170.5, 197.0, and 246.5 mg/dL, *P* = 0.021 for t-chol; 97.5, 117.0, and 152.0 mg/dL, *P* = 0.036 for LDL-C) (Table 3). Both t-chol and LDL-C levels correlated with lobular inflammation (rs = 0.304, *P* = 0.015, and rs = 0.264, *P* = 0.036, respectively).

In addition, the difference in the proportion of subjects with MetS was marginally significant with the grade of lobular inflammation (28.1, 48.3, and 100%, respectively, *P* = 0.050, *P* for linear trend = 0.038) (Table 3).

However, t-chol, LDL-C, and MetS were not significantly affected by portal inflammation (Table 6). Furthermore, other metabolic parameters showed no significant difference according to the grade of ballooning degeneration and the stage of hepatic fibrosis (Tables 4, 5).

**Table 5:** Comparison of demographic, anthropometric, laboratory, and magnetic resonance imaging-measured fat fraction findings of 63 children with non-alcoholic fatty liver disease by liver histologic fibrosis stage.

**Variable**	**Hepatic fibrosis Stage 0** **(*n* = 11)**	**Hepatic fibrosis Stage 1** **(*n* = 41)**	**Hepatic fibrosis** **Stage 2** **(*n* = 11)**	***P*-value**
Sex (boys: girls)	10 (90.9%): 1 (9.1%)	32 (78.0%): 9 (22.0%)	6 (54.5%): 5 (45.5%)	0.172^*^
Age, yr	12.2 (5.6–17.4)	12.6 (6.1–19.3)	12.6 (9.9–18.1)	0.907
Height (cm)	153.7 (122.4–183.0)	152.6 (123.8–184.7)	156.5 (144.3–184.5)	0.824
Height *z*-score	1.02 (−0.57–1.95)	0.85 (−3.40–2.17)	0.89 (−2.90–2.82)	0.686
Weight (kg)	63.0 (37.9–112.7)	69.3 (36.4–130.9)	64.6 (45.2–117.5)	0.989
Weight *z*-score	2.43 (0.41–4.19)	2.16 (−2.80–5.01)	1.86 (−0.32–4.18)	0.519
WC (cm)	87.0 (81.0–113.2)	90.0 (67.0–120.0)	88.3 (80.0–110.0)	0.774
WHtR	0.60 (0.52–0.69)	0.60 (0.46–0.69)	0.56 (0.53–0.66)	0.338
BMI	26.7 (22.6–38.3)	27.5 (18.6–38.4)	26.1 (21.2–34.5)	0.633
BMI *z*-score	2.31 (0.89–4.93)	2.62 (−1.10–5.10)	1.63 (0.38–4.07)	0.441
Fat mass (kg)	22.8 (17.5–46.0)	24.4 (8.2–54.0)	20.6 (14.5–35.3)	0.533
Fat mass (%)	36.3 (29.8–41.5)	36.1 (22.3–67.2)	33.1 (22.5–45.1)	0.487
FFM (kg)	46.4 (27.8–74.7)	40.3 (23.5–76.3)	46.0 (29.4–79.7)	0.583
FFM (%)	63.8 (58.5–70.2)	63.9 (32.8–77.7)	67.0 (54.8–77.5)	0.475
FMI	9.4 (7.9–16.0)	10.3 (3.9–18.8)	8.2 (5.5–13.2)	0.324
FFMI	17.8 (13.2–22.6)	16.5 (9.2–22.9)	18.0 (13.2–23.4)	0.820
mSBP (mmHg)	115.0 (100–159)	120.0 (100–148)	114.0 (105–136)	0.304
mDBP (mmHg)	63.0 (53–90)	66.0 (52–84)	61.0 (52–74)	0.251
AST (IU/L)	46.0 (29–74)	57.0 (17–216)	61.0 (27–226)	0.390
ALT (IU/L)	98.0 (23–187)	126.0 (21–342)	122.0 (20–366)	0.414
AST/ALT	0.44 (0.37–1.26)	0.48 (0.28–1.52)	0.52 (0.29–1.45)	0.815
Total bilirubin (mg/dL)	0.5 (0.3–1.1)	0.6 (0.2–1.3)	0.6 (0.3–1.1)	0.816
GGT (IU/L)	29.0 (12–101)	38.0 (12–133)	50.0 (21–184)	0.151
Total cholesterol (mg/dL)	169.0 (139–230)	177.0 (105–275)	201.0 (122–277)	0.581
Triglyceride (mg/dL)	101.0 (73–251)	122.0 (54–357)	130.0 (48–364)	0.643
HDL-C (mg/dL)	44.0 (36–58)	46.0 (28–68)	41.0 (28–53)	0.415
LDL-C (mg/dL)	94.0 (79–147)	106.0 (55–175)	120.0 (61–168)	0.623
FPG (mg/dL)	90.0 (84–119)	97.0 (78–270)	91.0 (71–153)	0.250
Insulin (mIU/L)	20.6 (4.6–51.2)	22.4 (8.9–75.6)	16.9 (6.4–37.1)	0.421
HbA1c (%)	5.1 (5.0–5.7)	5.4 (5.1–13.5)	5.5 (5.0–10.5)	0.140
HOMA-IR	4.5 (1.0–12.8)	6.0 (2.0–17.0)	4.5 (2.1–7.9)	0.182
QUICKI	0.31 (0.27–0.39)	0.30 (0.26–0.34)	0.31 (0.29–0.34)	0.182
MRI HFF (%)	25.4 (6.4–46.2)	25.2 (4.7–49.9)	17.2 (9.0–31.5)	0.369
MRI PFF (%)	3.2 (1.6–14.8)	4.3 (1.0–26.9)	3.6 (0.4–15.8)	0.909
BMI: Normal/Overweight/Obesity (*n*, %)	1 (9.1%)/1 (9.1%)/9 (81.8%)	3 (7.3%)/7 (17.1%)/31 (75.6%)	1 (9.1%)/5 (45.5%)/5 (45.5%)	0.219^*^
Central obesity (*n*, %)	9 (81.8%)	36 (87.8%)	9 (81.8%)	0.655^*^
Normal BP/EBP + HTN (*n*, %)	7 (63.6%)/4 (36.4%)	23 (56.1%)/18 (43.9%)	8 (72.7%)/3 (27.3%)	0.654^*^
Dyslipidemia (*n*, %)	6 (54.5%)	27 (65.9%)	9 (81.8%)	0.393^*^
Normal/PreDM + DM (*n*, %)	7 (63.6%)/4 (36.4%)	25 (61.0%)/16 (39.0%)	8 (72.7%)/3 (27.3%)	0.927^*^
MetS (*n*, %)	4 (36.4%)	15 (36.6%)	6 (54.5%)	0.564^*^

*P-value was calculated by Kruskal-Wallis test*.

* *P-value was analyzed by Fisher's exact test. Values are presented as median (range) or numbers (%)*.

*WC, waist circumference; WHtR, waist circumference-to-height ratio; BMI, body mass index; FFM, fat free mass; FMI, fat mass index = fat mass(kg)/height(m)^2^; FFMI, fat free mass index = FFM(kg)/height(m)^2^; mSBP, mean systolic blood pressure; mDBP, mean diastolic blood pressure; AST, aspartate aminotransferase; ALT, alanine aminotransferase; GGT, γ-glutamyl transferase; HDL-C, high density lipoprotein cholesterol; LDL-C, low density lipoprotein cholesterol; FPG, fasting plasma glucose; HbA1c, glycated hemoglobin; HOMA-IR, homeostatic model assessment of insulin resistance; QUICKI, Quantitative insulin sensitivity check index; MRI, magnetic resonance imaging; HFF, hepatic fat fraction; PFF, pancreatic fat fraction; BP, blood pressure; EBP, elevated BP; HTN, hypertension; DM, diabetes mellitus; PreDM, prediabetes; MetS, metabolic syndrome*.

**Table 6:** Comparison of demographic, anthropometric, laboratory, and magnetic resonance imaging-measured fat fraction findings of 63 children with non-alcoholic fatty liver disease by liver histologic portal inflammation grade.

**Variable**	**Portal inflammation** **Grade 0** **(*n* = 24)**	**Portal inflammation** **Grade 1–2** **(*n* = 39)**	***P*-value**
Sex (boys: girls)	19 (78.2%): 5 (20.8%)	29 (74.4%): 10 (25.6%)	0.663^*^
Age, yr	13.8 (5.6–19.3)	12.2 (6.1–18.2)	0.176
Height (cm)	156.1 (122.4–184.7)	155.8 (123.8–184.5)	0.318
Height *z*-score	1.0 (−1.78–2.17)	0.85 (−3.40–2.82)	0.697
Weight (kg)	75.8 (37.9–130.9)	64.6 (36.4–117.5)	0.123
Weight *z*-score	2.78 (−0.37–5.01)	1.96 (−2.80–4.18)	0.066
WC (cm)	99.9 (75.0–120.0)	88.0 (67.0–113.2)	0.015
WHtR	0.61 (0.50–0.69)	0.57 (0.46–0.67)	0.004
BMI	30.0 (20.0–38.4)	26.2 (18.6–38.3)	0.058
BMI *z*-score	3.36 (0–5.10)	2.40 (−1.10–4.93)	0.022
Fat mass (kg)	27.1 (12.9–52.4)	22.4 (8.2–54.0)	0.036
Fat mass (%)	37.5 (25.6–48.2)	34.9 (22.3–67.2)	0.165
FFM (kg)	42.7 (27.8–76.3)	40.0 (23.5–79.7)	0.179
FFM (%)	62.5 (51.8–74.4)	65.1 (32.8–77.7)	0.172
FMI	11.0 (5.8–17.1)	8.7 (3.9–18.8)	0.033
FFMI	18.2 (13.2–22.9)	16.3 (9.2–23.4)	0.172
mSBP (mmHg)	118.0 (100–150)	117.0 (100–159)	0.210
mDBP (mmHg)	65.0 (52–84)	63.0 (52–90)	0.205
AST (IU/L)	62.0 (25–226)	51.0 (17–190)	0.361
ALT (IU/L)	127.0 (20–342)	115.0 (21–366)	0.666
AST/ALT	0.48 (0.31–1.45)	0.49 (0.28–1.52)	0.246
Total bilirubin (mg/dL)	0.6 (0.2–1.3)	0.5 (0.3–1.1)	0.224
GGT (IU/L)	39.0 (12–184)	37.0 (12–158)	0.739
Total cholesterol (mg/dL)	179.0 (122–221)	180.0 (105–277)	0.488
Triglyceride (mg/dL)	129.0 (48–280)	118.0 (56–364)	0.462
HDL-C (mg/dL)	45.5 (28–68)	45.0 (28–67)	0.228
LDL-C (mg/dL)	113.0 (66–147)	106.0 (55–175)	0.506
FPG (mg/dL)	90.0 (71–270)	94.0 (71–179)	0.266
Insulin (mIU/L)	21.0 (4.6–75.4)	20.9 (6.4–75.6)	0.837
HbA1c (%)	5.4 (5.0–11.0)	5.3 (5.1–13.5)	0.964
HOMA-IR	4.9 (1.0–14.5)	5.1 (2.0–17.0)	0.932
QUICKI	0.30 (0.27–0.39)	0.30 (0.26–0.34)	0.932
MRI HFF (%)	23.4 (6.4–48.1)	26.3 (4.7–49.9)	0.192
MRI PFF (%)	4.0 (1.0–26.9)	3.8 (0.4–15.8)	0.887
BMI: Normal/Overweight/Obesity (*n*, %)	2 (8.3%)/3 (12.5%)/19 (79.2%)	3 (7.7%)/10 (25.6%)/26 (66.7%)	0.470^†^
Central obesity (*n*, %)	22 (91.7%)	32 (82.1%)	0.462^†^
Normal BP/EBP + HTN (*n*, %)	13 (54.2%)/11 (45.8%)	25 (64.1%)/14 (35.9%)	0.434^*^
Dyslipidemia (*n*, %)	16 (66.7%)	25 (64.1%)	0.836^*^
Normal/PreDM + DM (*n*, %)	16 (66.7%)/8 (33.3%)	24 (61.5%)/15 (38.5%)	0.681^*^
MetS (*n*, %)	12 (50.0%)	13 (33.3%)	0.189^*^

*P-value was calculated by Mann Whitney U-test*.

* *P-value was calculated by Chi-square test*.

† *P-value was calculated by Fisher's exact test. Values are presented as median (range) or numbers (%)*.

*WC, waist circumference; WHtR, waist circumference-to-height ratio; BMI, body mass index; FFM, fat free mass; FMI, fat mass index = fat mass(kg)/height(m)^2^; FFMI, fat free mass index = FFM(kg)/height(m)^2^; mSBP, mean systolic blood pressure; mDBP, mean diastolic blood pressure; AST, aspartate aminotransferase; ALT, alanine aminotransferase; GGT, γ-glutamyl transferase; HDL-C, high density lipoprotein cholesterol; LDL-C, low density lipoprotein cholesterol; FPG, fasting plasma glucose; HbA1c, glycated hemoglobin; HOMA-IR, homeostatic model assessment of insulin resistance; QUICKI, Quantitative insulin sensitivity check index; MRI, magnetic resonance imaging; HFF, hepatic fat fraction; PFF, pancreatic fat fraction; BP, blood pressure; EBP, elevated BP; HTN, hypertension; DM, diabetes mellitus; PreDM, prediabetes; MetS, metabolic syndrome*.

## Discussion

To our knowledge, the present study is the first to evaluate the clinical findings, metabolic parameters, and MRI-measured fractions of ectopic fat using the histopathological findings of the liver in children with NAFLD. We highlight the following findings. MRI-measured HFF was associated with histological steatosis but was not associated with lobular inflammation, hepatocyte ballooning, and hepatic fibrosis in children with NAFLD. MRI-measured PFF was not associated with any histological features of the liver. Although some obesity-related factors were associated with the grade of steatosis, liver enzymes (AST and ALT) were associated with lobular inflammation. Lobular inflammation was also associated with elevated serum levels of t-chol and LDL-C in children with NAFLD, while BMI *z*-score, TBF percentage, and MRI-measured HFF and PFF showed no significant differences based on the grade of lobular inflammation.

In our study, MRI-measured HFF was correlated only with the grade of hepatic steatosis among the histological criteria of NAFLD. This suggests that the severity of hepatic steatosis is not necessarily proportional to the severity of hepatic inflammation. A recent meta-analysis showed that MRI-PDFF of the liver had an excellent diagnostic value for the assessment of hepatic fat content and the classification of histologic steatosis in patients with NAFLD (26). In particular, it is known that the correlation between MRI-estimated hepatic fat and histologically-determined steatosis is better in patients with no or mild hepatic fibrosis than in patients with moderate or severe fibrosis because of the reduction in the number of hepatocytes due to replacement by fibrosis (27, 28). In line with our findings, Bril et al. (29) showed that histological severity of NAFLD, including inflammation, ballooning, and fibrosis, was not associated with the amount of intrahepatic fat content measured by MRS. They suggested that histological activity appears to have an early threshold of about 6 ± 2% and is not significantly influenced by the increasing amounts of intrahepatic TG accumulation beyond this threshold (29). Another study on adults with NAFLD showed that MRI-PDFF of the liver positively correlated with the grade of histologic steatosis and NAS and did not correlate with the grade of ballooning or lobular inflammation, which is similar to the results of our study (30). However, this study on adults showed that MRI-PDFF of the liver negatively correlated with the stage of fibrosis (30), while our study in pediatric NAFLD patients revealed no correlation between HFF and stage of fibrosis. Another study on adult patients with NAFLD showed that MRI-PDFF findings of the liver showed no statistically significant difference between fibrosis stages 0–2 and stages 3–4 (31). In another study, repeat MRI-PDFF was performed to assess hepatic steatosis, and magnetic resonance elastography (MRE) was performed to assess hepatic fibrosis; both MRI examinations were performed simultaneously in children with NAFLD over a mean follow-up of 27 months (32). The study showed that there was no significant correlation between the changes in MRI-measured HFF and those in MRE-measured hepatic stiffness (32). Some interventional studies on adult patients with NAFLD have shown significant improvement in the histologic grade of steatosis or on MRI-PDFF of the liver without any changes in inflammation, ballooning, or fibrosis (33, 34). However, only a few studies have shown that hepatic steatosis is associated with other histological features of NAFLD. Idilman et al. (35) showed a correlation between the histological grade of steatosis and the grade of necroinflammation. Ajmera et al. (36) showed that a higher liver MRI-PDFF at baseline was associated with the progression of fibrosis after a median follow-up of 1.75 years in adults with NAFLD.

Our study revealed that PFF was neither correlated with MRI-measured HFF nor the histological steatosis grade of NAFLD, suggesting that the pathophysiology contributing to pancreatic fat might be different from that of hepatic fat, although both represent ectopic fat accumulation in patients with obesity. Based on previous studies, the association between pancreatic and hepatic steatosis remains inconsistent in patients with NAFLD. To date, there have been only a few studies on adults and no study based on the histopathological findings in pediatric patients. Patel et al. (37) showed that MRI-determined PFF significantly increased with increasing grade of histological steatosis in adults with NAFLD. In contrast, Idilman et al. (35) showed no correlation between liver MRI-PDFF or histological steatosis grade and pancreatic MRI-PDFF in adults with NAFLD.

Regarding the relations between liver enzymes and histological features of the liver or MRI findings, the results from our study revealed that serum AST and ALT levels significantly increased with increasing lobular inflammation but not with histological steatosis or MRI-measured HFF. Since elevated liver enzymes represent hepatic inflammation, this result also verifies that hepatic inflammation is not caused by hepatic steatosis alone. The association between liver enzymes and histological features in NAFLD remains unclear. Idilman et al. (35) showed that in adults with biopsy-proven NAFLD, there was a positive correlation between histological steatosis and laboratory markers, including AST, ALT, and total bilirubin, as well as between histological steatosis and the grade of necroinflammation on liver histopathology. Meanwhile, Bril et al. (29) showed that regardless of the lack of association between MRS-measured intrahepatic fat and the severity of liver histology, measured by inflammation, ballooning, and fibrosis, the increase in intrahepatic fat content was associated with a linear increase in ALT and AST levels. Permutt et al. (38) showed that adult NAFLD patients with steatosis grade 1 might display characteristics of advanced liver disease (higher average AST/ALT ratio, GGT, and higher stage of fibrosis and hepatocellular ballooning), rather than those with grade 3 steatosis, highlighting that a low HFF does not necessarily indicate mild NAFLD. Schwimmer et al. (39) reported a weak but significant correlation between ALT and MRE-measured liver stiffness as a biomarker of hepatic fibrosis in children with biopsy-proven NAFLD. However, Mouzaki et al. (32) found no correlation between changes in MRE-measured liver stiffness and ALT changes, and only a trend toward a weak correlation between changes in ALT and changes in MRI-PDFF measured-HFF. In a previous study on pediatric patients, the entire spectrum of histologic features of NAFLD, including advanced fibrosis, was observed even in children with normal liver enzymes (40).

In our study, as the grade of lobular inflammation increased, t-chol and LDL-C significantly increased, although obesity-related factors, such as BMI *z*-score, TBF percentage, and MRI-measured ectopic fat, were not significantly different among lobular inflammation grades. MRI-measured HFF or the histological grade of steatosis was not correlated with any components of the lipid profile. We suggest that hepatic inflammation in NAFLD might have an adverse effect on lipid metabolism. Bril et al. (29) showed that neither plasma TG nor HDL-C levels were correlated with MRS-measured intrahepatic fat content beyond an intrahepatic fat content of >8%. Idilman et al. (35) showed a negative correlation between histological steatosis or MRI-measured HFF and HDL-C, which could be due to a significant correlation between the histologic steatosis and necroinflammation. Mouzaki et al. (32) showed that changes in MRI-measured HFF were inversely correlated with changes in serum HDL-C levels; however, changes in MRE-measured liver stiffness as a surrogate biomarker of hepatic fibrosis did not correlate with any changes in the lipid profiles.

Our study included 18 (28.6%) subjects with non-obese NAFLD; of these, 13 (20.6%) were overweight (the 85th ≤ BMI <95th percentiles), and 5 (7.9%) were within the normal range (BMI <85th percentile). A systematic meta-analysis showed the prevalence of NAFLD in children and adolescents with normal weight to be 2.3% (95% CI, 1.5–3.6), in subjects with overweight to be 12.5% (95% CI, 9.2–16.7), and that in subjects with obesity to be 36.1% (95% CI, 24.6–49.4) in studies conducted among the general population (41). Non-obese NAFLD who may be overweight (BMI 85th−95th percentile adjusted for age and sex) tend to be younger, male, and have lower BP, FPG, HbA1c levels, and genetic polymorphisms (42). Until now, there are no published studies on the histological features of pediatric non-obese NAFLD (42). In one study wherein the main objective was to assess the hepatic iron content in children with biopsy-proven NAFLD, normal weight children with NAFLD did not show significant differences in laboratory parameters and liver histology compared to overweight or obese children with NAFLD (42, 43). In our study, the comparison between the non-obese (*n* = 18) and obese groups (*n* = 45) with NAFLD did not show significant differences in liver enzymes, metabolic parameters (Supplementary Table 1), and liver histological features except for hepatic steatosis (Supplementary Table 2). The number of non-obese patients with NAFLD might have been insufficient to analyze the differences between the two groups. More studies are required to identify the differences between non-obese NAFLD and obese NAFLD.

The present study has some limitations. First, children showing severe histological findings are relatively uncommon. Among 63 children, two had grade 3 lobular inflammation; only one had grade 2 portal inflammation, and there were no children with stage 3–4 fibrosis. This might be because children with NAFLD tend to have a relatively shorter duration of the disease than adults with NAFLD; thus, pediatric NAFLD with severe histological findings is generally infrequent. Second, we did not perform MRE to compare the severity of fibrosis using MRI modalities with histologic findings of fibrosis. Identification of fibrosis in children with NAFLD is important because fibrosis is more likely to progress to cirrhosis, hepatocellular carcinoma, and liver-related mortality (2, 9, 44). To date, MRE has predominantly been performed in adults. However, surrogate quantitative imaging biomarkers, including MRE, are necessary for indirect assessment of hepatic fibrosis because repeated liver biopsy over a short-term period is impractical due to its invasive nature, the potential for sampling error, and reluctance to biopsy (2). A few studies conducted in children have assessed hepatic fibrosis using MRE (32, 39, 45–47). Further validation studies using MRE are warranted to determine the optimal cut-off points and the ability to assess fibrosis in children with NAFLD longitudinally (2).

Third, the retrospective cross-sectional design and relatively small sample size used in this study warrant further large-scale prospective longitudinal studies in the future to assess the association between MRI-measured ectopic fat content, histological findings, and metabolic changes, especially in pediatric patients with NAFLD.

In conclusion, the present study showed that hepatic steatosis on MRI was only associated with the grade of liver steatosis on histopathology. The increase in hepatic steatosis (MRI-measured HFF or histologic steatosis grade) was not correlated with other histological features of NAFLD, including lobular inflammation, hepatocyte ballooning, and hepatic fibrosis. In addition, MRI-measured pancreatic fat as additional obesity-related ectopic fat was not related to any histologic features of the liver. Instead, elevated serum liver enzymes (AST and ALT) and some lipid profiles (t-chol, LDL-C) were associated with lobular inflammation. This study shows the importance of interpreting MRI in conjunction with anthropometric and laboratory findings of obesity and obesity-related complications in assessing pediatric patients suspected of NAFLD.

## Data Availability Statement

The original contributions presented in the study are included in the article/Supplementary Material, further inquiries can be directed to the corresponding author.

## Ethics Statement

This study was approved by the Institutional Review Board of Seoul National University Bundang Hospital (IRB No. B-2103-670-104). Written informed consent from the participants' legal guardian/next of kin was not required to participate in this study in accordance with the institutional requirements.

## Author Contributions

EL conducting the study, collecting and interpreting data, and drafting the manuscript. HY planning the study, interpreting data, and drafting the manuscript. JK conducting the study, interpreting data, and drafting the manuscript. All authors contributed to the article and approved the submitted version.

## Conflict of Interest

The authors declare that the research was conducted in the absence of any commercial or financial relationships that could be construed as a potential conflict of interest.

## Abbreviations

ALT: alanine aminotransferase
AST: aspartate aminotransferase
BMI: body mass index
BP: blood pressure
DBP: diastolic blood pressure
DM: diabetes mellitus
EBP: elevated blood pressure
FFM: fat-free mass
FFMI: fat-free mass index
FMI: fat mass index
FPG: fasting plasma glucose
GGT: γ-glutamyl transferase
HbA1c: glycated hemoglobin
HDL-C: high-density lipoprotein cholesterol
HFF: hepatic fat fraction
HOMA-IR: homeostatic model assessment of insulin resistance
HTN: hypertension
LDL-C: low-density lipoprotein cholesterol
MetS: metabolic syndrome
MRE: magnetic resonance elastography
MRI: magnetic resonance imaging
MRS: magnetic resonance spectroscopy
NAFLD: non-alcoholic fatty liver disease
NAS: non-alcoholic fatty liver disease activity score
NASH: non-alcoholic steatohepatitis
PDFF: proton density fat fraction
PFF: pancreatic fat fraction
PreDM: prediabetes
QUICKI: quantitative insulin sensitivity check index
rs: Spearman's rho coefficient
SBP: systolic blood pressure
TBF: total body fat
t-bil: total bilirubin
t-chol: total cholesterol
TG: triglyceride
WC: waist circumference
WHtR: waist circumference to height ratio.

## References

[1] Yang HR Kim HR Kim MJ Ko JS Seo JK. Noninvasive parameters and hepatic fibrosis scores in children with nonalcoholic fatty liver disease. World J Gastroenterol. (2012) 18:1525–30. 10.3748/wjg.v18.i13.1525 22509085 PMC3319949

[2] Vos MB Abrams SH Barlow SE Caprio S Daniels SR Kohli R. NASPGHAN clinical practice guideline for the diagnosis and treatment of nonalcoholic fatty liver disease in children: recommendations from the Expert Committee on NAFLD (ECON) and the North American Society of Pediatric Gastroenterology, Hepatology and Nutrition (NASPGHAN). J Pediatr Gastroenterol Nutr. (2017) 64:319–34. 10.1097/MPG.0000000000001482 28107283 PMC5413933

[3] Kleiner DE Brunt EM. Nonalcoholic fatty liver disease: pathologic patterns and biopsy evaluation in clinical research. Semin Liver Dis. (2012) 32:3–13. 10.1055/s-0032-1306421 22418883

[4] Romana BS Chela H Dailey FE Dailey FE Nassir F Tahan V. Non-alcoholic fatty pancreas disease (NAFPD): a silent spectator or the fifth component of metabolic syndrome? A literature review. Endocr Metab Immune Disord Drug Targets. (2018) 18:547–54. 10.2174/1871530318666180328111302 29595117

[5] Yu TY Wang CY. Impact of non-alcoholic fatty pancreas disease on glucose metabolism. J Diabetes Investig. (2017) 8:735–47. 10.1111/jdi.12665 PMC5668526 28371475

[6] Majumder S Philip NA Takahashi N Levy MJ Singh VP Chari ST. Fatty pancreas: should we be concerned? Pancreas. (2017) 46:1251–8. 10.1097/MPA.0000000000000941 29040194 PMC6689238

[7] Singh RG Yoon HD Wu LM Lu J Plank LD Petrov MS. Ectopic fat accumulation in the pancreas and its clinical relevance: a systematic review, meta-analysis, and meta-regression. Metabolism. (2017) 69:1–13. 10.1016/j.metabol.2016.12.012 28285638

[8] Ko JS. New perspectives in pediatric nonalcoholic fatty liver disease: epidemiology, genetics, diagnosis, and natural history. Pediatr Gastroenterol Hepatol Nutr. (2019) 22:501–10. 10.5223/pghn.2019.22.6.501 31777715 PMC6856496

[9] Angulo P Kleiner DE Dam-Larsen S Adams LA Bjornsson ES Charatcharoenwitthaya P. Liver fibrosis, but no other histologic features, is associated with long-term outcomes of patients with nonalcoholic fatty liver disease. Gastroenterology. (2015) 149:389–97.e10. 10.1053/j.gastro.2015.04.043 25935633 PMC4516664

[10] Schwimmer JB Middleton MS Behling C Newton KP Awai HI Paiz MN. Magnetic resonance imaging and liver histology as biomarkers of hepatic steatosis in children with nonalcoholic fatty liver disease. Hepatology. (2015) 61:1887–95. 10.1002/hep.27666 25529941 PMC4670559

[11] Lee MJ Bagci P Kong J Vos MB Sharma P Kalb B. Liver steatosis assessment: correlations among pathology, radiology, clinical data and automated image analysis software. Pathol Res Pract. (2013) 209:371–9. 10.1016/j.prp.2013.04.001 23707550

[12] Tang A Desai A Hamilton G Wolfson T Gamst A Lam J. Accuracy of MR imaging-estimated proton denstiy fat fraction for classification of dichotomized histologic steatosis grades in nonalcoholic fatty liver disease. Radiology. (2015) 274:416–25. 10.1148/radiol.14140754 25247408 PMC4314291

[13] Faul F Erdfelder E Lang AG Buchner A. G^*^Power 3: a flexible statistical power analysis program for the social, behavioral, and biomedical sciences. Behav Res Methods. (2007) 39:175–91. 10.3758/bf03193146 17695343

[14] Holland-Hall CM. Chapter 132. Adolescent physical and social development. In: Robert MK Joseph St. G, editors. Nelson Textbook of Pediatrics. 21st ed. Philadelphia, PA: Elsevier Press (2019). p. 1014–20.

[15] Kim JH Yun S Hwang SS Shim JO Chae HW Lee YJ. The 2017 Korean national growth charts for children and adolescents: development, improvement, and prospects. Korean J Pediatr. (2018) 61:135–49. 10.3345/kjp.2018.61.5.135 29853938 PMC5976563

[16] National Institute of Health. 2007 Korean National Growth Charts [in Korean]. (2008). Available online at: http://www.nih.go.kr/board/board.es?mid=a40801000000&bid=0050&act=view&list_no=1235 (accessed March 10, 2021).

[17] Flynn JT Kaelber DC Baker-Smith CM Blowey D Carroll A Daniels SR. Clinical practice guideline for screening and management of high blood pressure in children and adolescents. Pediatrics. (2017) 140:e20171904. 10.1542/peds.2017-1904 28827377

[18] Weaver DJ Jr. Pediatric hypertension: review of updated guidelines. Pediatr Rev. (2019) 40:354–8. 10.1542/pir.2018-0014 31263043

[19] Conwell LS Trost SG Brown WJ Batch JA. Indexes of insulin resistance and secretion in obese children and adolescents: a validation study. Diabetes Care. (2004) 27:314–9. 10.2337/diacare.27.2.314 14747206

[20] American Diabetes Association. Diagnosis and classification of diabetes mellitus. Diabetes Care. (2013) 36(Suppl. 1):S67–74. 10.2337/dc13-S067 23264425 PMC3537273

[21] Expert Panel on Integrated Guidelines for Cardiovascular Health and Risk Reduction in Children and Adolescents National Heart Lung and Blood Institute. Expert panel on integrated guidelines for cardiovascular health and risk reduction in children and adolescents: summary report. Pediatrics. (2011) 128(Suppl. 5):S213–56. 10.1542/peds.2009-2107C 22084329 PMC4536582

[22] Ford ES Ajani UA Mokdad AH National Health and Nutrition Examination. The metabolic syndrome and concentrations of C-reactive protein among U.S. youth. Diabetes Care. (2005) 28:878–81. 10.2337/diacare.28.4.878 15793189

[23] Cook S Weitzman M Auinger P Nguyen M Dietz WH. Prevalence of a metabolic syndrome phenotype in adolescents: findings from the third national health and nutrition examination survey, 1988–1994. Arch Pediatr Adolesc Med. (2003) 157:821–7. 10.1001/archpedi.157.8.821 12912790

[24] Kleiner DE Brunt EM Van Natta M Behling C Contos MJ Cummings OW. Design and validation of a histological scoring system for nonalcoholic fatty liver disease. Hepatology. (2005) 41:1313–21. 10.1002/hep.20701 15915461

[25] Brunt EM Kleiner DE Wilson LA Belt P Neuschwander-Tetri BA NASH Clinical Research Network. Nonalcoholic fatty liver disease (NAFLD) activity score and the histopathologic diagnosis in NAFLD: distinct clinicopathologic meanings. Hepatology. (2011) 53:810–20. 10.1002/hep.24127 21319198 PMC3079483

[26] Gu J Liu S Du S Zhang Q Xiao J Dong Q. Diagnostic value of MRI-PDFF for hepatic steatosis in patients with non-alcoholic fatty liver disease: a meta-analysis. Eur Radiol. (2019) 29:3564–73. 10.1007/s00330-019-06072-4 30899974

[27] Idilman IS Keskin O Celik A Savas B Elhan AH Idilman R. A comparison of liver fat content as determined by magnetic resonance imaging-proton density fat fraction and MRS versus liver histology in non-alcoholic fatty liver disease. Acta Radiol. (2016) 57:271–8. 10.1177/0284185115580488 25855666

[28] McPherson S Jonsson JR Cowin GJ O'Rourke P Clouston AD Volp A. Magnetic resonance imaging and spectroscopy accurately estimate the severity of steatosis provided the stage of fibrosis is considered. J Hepatol. (2009) 51:389–97. 10.1016/j.jhep.2009.04.012 19505740

[29] Bril F Barb D Portillo-Sanchez P Biernacki D Lomonaco R Suman A. Metabolic and histological implications of intrahepatic triglyceride content in nonalcoholic fatty liver disease. Hepatology. (2017) 65:1132–44. 10.1002/hep.28985 27981615

[30] Wildman-Tobriner B Middleton MM Moylan CA Rossi S Flores O Chang ZA. Association between magnetic resonance imaging-proton density fat fraction and liver histology features in patients with nonalcoholic fatty liver disease or nonalcoholic steatohepatitis. Gastroenterology. (2018) 155:1428–35.e2. 10.1053/j.gastro.2018.07.018 30031769 PMC6456892

[31] Lin SC Heba E Bettencourt R Lin GY Valasek MA Lunde O. Assessment of treatment response in non-alcoholic steatohepatitis using advanced magnetic resonance imaging. Aliment Pharmacol Ther. (2017) 45:844–54. 10.1111/apt.13951 28116801 PMC5346270

[32] Mouzaki M Trout AT Arce-Clachar AC Bramlage K Kuhnell P Dillman JR. Assessment of nonalcoholic fatty liver disease progression in children using magnetic resonance imaging. J Pediatr. (2018) 201:86–92. 10.1016/j.jpeds.2018.05.024 30041934 PMC6429948

[33] Ratziu V Giral P Jacqueminet S Charlotte F Hartemann-Heurtier A Serfaty L. Rosiglitazone for nonalcoholic steatohepatitis: one-year results of the randomized placebo-controlled fatty liver improvement with rosiglitazone therapy (FLIRT) trial. Gastroenterology. (2008) 135:100–10. 10.1053/j.gastro.2008.03.078 18503774

[34] Noureddin M Lam J Peterson MR Middleton M Hamilton G Le TA. Utility of magnetic resonance imaging versus histology for quantifying changes in liver fat in nonalcoholic fatty liver disease trials. Hepatology. (2013) 58:1930–40. 10.1002/hep.26455 23696515 PMC4819962

[35] Idilman IS Tuzun A Savas B Elhan AH Celik A Idilman R. Quantification of liver, pancreas, kidney, and vertebral body MRI-PDFF in non-alcoholic fatty liver disease. Abdom Imaging. (2015) 40:1512–9. 10.1007/s00261-015-0385-0 25715922

[36] Ajmera V Park CC Caussy C Singh S Hernandez C Bettencourt R. Magnetic resonance imaging proton density fat fraction associates with progression of fibrosis in patients with nonalcoholic fatty liver disease. Gastroenterology. (2018) 155:307–10.e2. 10.1053/j.gastro.2018.04.014 29660324 PMC6090543

[37] Patel NS Peterson MR Brenner DA Heba E Sirlin C Loomba R. Association between novel MRI-estimated pancreatic fat and liver histology-determined steatosis and fibrosis in non-alcoholic fatty liver disease. Aliment Pharmacol Ther. (2013) 37:630–9. 10.1111/apt.12237 23383649 PMC4136524

[38] Permutt Z Le TA Peterson MR Seki E Brenner DA Sirlin C. Correlation between liver histology and novel magnetic resonance imaging in adult patients with non-alcoholic fatty liver disease - MRI accurately quantifies hepatic steatosis in NAFLD. Aliment Pharmacol Ther. (2012) 36:22–9. 10.1111/j.1365-2036.2012.05121.x 22554256 PMC3437221

[39] Schwimmer JB Behling C Angeles JE Paiz M Durelle J Africa J. Magnetic resonance elastography measured shear stiffness as a biomarker of fibrosis in pediatric nonalcoholic fatty liver disease. Hepatology. (2017) 66:1474–85. 10.1002/hep.29241 28493388 PMC5650504

[40] HH AK Henderson J Vanhoesen K Ghishan F Bhattacharyya A. Nonalcoholic fatty liver disease in children: a single center experience. Clin Gastroenterol Hepatol. (2008) 6:799–802. 10.1016/j.cgh.2008.03.001 18486560

[41] Anderson EL Howe LD Jones HE Higgins JP Lawlor DA Fraser A. The prevalence of non-alcoholic fatty liver disease in children and adolescents: a systematic review and meta-analysis. PLoS ONE. (2015) 10:e0140908. 10.1371/journal.pone.0140908 26512983 PMC4626023

[42] Wang AY Dhaliwal J Mouzaki M. Lean non-alcoholic fatty liver disease. Clin Nutr. (2019) 38:975–81. 10.1016/j.clnu.2018.08.008 30466956

[43] Manco M Alisi A Real JF Equitani F DeVito R Valenti L. Early interplay of intra-hepatic iron and insulin resistance in children with non-alcoholic fatty liver disease. J Hepatol. (2011) 55:647–53. 10.1016/j.jhep.2010.12.007 21168460

[44] Loomba R Chalasani N. The hierarchial model of NAFLD: prognostic significance of histologic features in NASH. Gastroenterology. (2015) 149:278–81. 10.1053/j.gastro.2015.06.016 26116800

[45] Xanthakos SA Podberesky DJ Serai SD Miles L King EC Balistreri WF. Use of magnetic resonance elastography to assess hepatic fibrosis in children with chronic liver disease. J Pediatr. (2014) 164:186–8. 10.1016/j.jpeds.2013.07.050 24064151 PMC3872246

[46] Etchell E Juge L Hatt A Sinkus R Bilston LE. Liver stiffness values are lower in pediatric subjects than in adults and increase with age: a multifrequency MR elastography study. Radiology. (2017) 283:222–30. 10.1148/radiol.2016160252 27755913

[47] Goyal NP Sawh MC Ugalde-Nicalo P Angeles JE Proudfoot JA Newton KP. Evaluation of quantitative imaging biomarkers for early-phase clinical trials of steatohepatitis in adolescents. J Pediatr Gastroenterol Nutr. (2020) 70:99–105. 10.1097/MPG.0000000000002535 31633654 PMC8053386

