# Supplementary material for: Relationship Between Histological Features of Non-alcoholic Fatty Liver Disease and Ectopic Fat on Magnetic Resonance Imaging in Children and Adolescents
Source: Front Pediatr. 2021 Jun 10;9:685795. doi: 10.3389/fped.2021.685795 (PMC8222518; doi:10.3389/fped.2021.685795)
Supplement: Supplementary file 1 [file Table_1.pdf]

Supplementary Table 1. Comparison of demographic, anthropometric, laboratory, and magnetic resonance imaging-measured fat fraction findings between non-obese NAFLD and obese NAFLD

	BMI < 95th percentile (n = 18)	BMI ≥ 95th percentile (n = 45)	*P*-value
Sex (boys : girls)	13 (72.2%) : 5 (27.8%)	35 (77.8%) :10 (22.2%)	0.746*
Age, yr	12.3 (9.2–18.2)	12.7 (5.6–19.3)	0.648
Height (cm)	148.9 (138.2–177.5)	157.6 (122.4–184.7)	0.146
Height z-score	0.36 (-3.40–1.68)	0.98 (-1.78–2.82)	0.033
Weight (kg)	49.6 (39.2–82.5)	72.8 (36.4–130.9)	< 0.001
Weight z-score	1.14 (-2.80–1.96)	2.60 (0.99–5.01)	< 0.001
WC (cm)	81.5 (67.0–90.0)	94.0 (75.0–120.0)	< 0.001
WHtR	0.53 (0.46–0.60)	0.61 (0.52–0.69)	< 0.001
BMI	22.6 (18.6–26.2)	28.9 (23.8–38.4)	< 0.001
BMI z-score	1.39 (-1.10–1.66)	2.93 (1.62–5.10)	< 0.001
Fat mass (kg)	17.3 (8.2–26.1)	26.4 (12.6–54.0)	< 0.001
Fat mass (%)	33.0 (22.3–45.1)	37.2 (24.1–67.2)	0.001
FFM (kg)	31.9 (27.6–59.6)	42.9 (23.5–79.7)	0.019
FFM (%)	67.1 (54.8–77.7)	62.7 (32.8–75.8)	0.001
FMI	7.2 (3.9–10.9)	10.7 (6.2–18.8)	< 0.001
FFMI	14.7 (13.2–19.0)	18.1 (9.2–23.4)	0.001
mSBP (mmHg)	113.7 (100–128)	120.0 (106–159)	0.008
mDBP (mmHg)	62.8 (52–74)	63.7 (52–90)	0.115
AST (IU/L)	49.0 (21–226)	58.0 (17–216)	0.447
ALT (IU/L)	97.5 (20–331)	128.0 (22–366)	0.155
AST/ALT	0.53 (0.37–1.52)	0.47 (0.28–1.26)	0.086
Total bilirubin (mg/dL)	0.6 (0.3–1.1)	0.5 (0.2–1.3)	0.866
GGT (IU/L)	35.5 (12–184)	38.0 (12–158)	0.732
Total cholesterol (mg/dL)	179.5 (120–277)	180.0 (105–275)	0.377
Triglyceride (mg/dL)	105.0 (48–364)	124.0 (54–293)	0.681
HDL-C (mg/dL)	43.5 (37–67)	46.0 (28–68)	0.964
LDL-C (mg/dL)	101.0 (61–168)	112.0 (55–175)	0.198
FPG (mg/dL)	92.5 (76–153)	94.0 (71–270)	0.867
Insulin (mIU/L)	18.2 (4.6–46.3)	22.4 (6.4–75.6)	0.104
HbA1c (%)	5.3 (5.0–7.7)	5.4 (5.0–13.5)	0.711
HOMA-IR	4.2 (1.0–14.0)	5.9 (2.0–17.0)	0.121
QUICKI	0.31 (0.27–0.39)	0.30 (0.26–0.34)	0.121
MRI HFF (%)	21.8 (4.7–33.0)	25.2 (6.4–49.9)	0.077
MRI PFF (%)	3.0 (0.4–15.8)	4.3 (1.0–26.9)	0.359
Central obese (n, %)	11 (61.1%)	43 (95.6%)	0.001*
Normal BP / EBP + HTN (n, %)	13 (72.2%) / 5 (27.8%)	25 (55.6%) / 20 (44.4%)	0.222†
Dyslipidemia (n, %)	10 (55.6%)	32 (71.1%)	0.237†
Normal / PreDM + DM (n, %)	11 (61.1%) / 7 (38.9%)	29 (64.4%) / 16 (35.6%)	0.804†
MetS (n, %)	4 (22.2%)	21 (46.7%)	0.073†

P-value was calculated by Mann Whitney *U* test. **P*-value was calculated by Fisher's exact test. †*P*-value was calculated by *Chi*-square test. Values are presented as median (range) or numbers (%).

WC, waist circumference; WHtR, waist circumference-to-height ratio; BMI, body mass index; FFM, fat-free mass; FMI, fat mass index = fat mass(kg)/height(m)^2; FFMI, fat-free mass index = FFM(kg)/ height(m)^2; mSBP, mean systolic blood pressure; mDBP, mean diastolic blood pressure; AST, aspartate aminotransferase; ALT, alanine aminotransferase; GGT, γ-glutamyl transferase; HDL-C, high density lipoprotein cholesterol; LDL-C, low density lipoprotein cholesterol; FPG, fasting plasma glucose; HbA1c, glycated hemoglobin; HOMA-IR, homeostatic model assessment of insulin resistance; QUICKI, Quantitative insulin sensitivity check index; MRI, magnetic resonance imaging; HFF, hepatic fat fraction; PFF, pancreatic fat fraction; BP, blood pressure; EBP, elevated BP; HTN, hypertension; DM, diabetes mellitus; PreDM, prediabetes; MetS, metabolic syndrome

Supplementary Table 2. Comparison of proportion of histologic steatosis, inflammation, ballooning and fibrosis between non-obese NAFLD and obese NAFLD

	Non-obese NAFLD (n = 18)	Obese NAFLD (n = 45)	Total (n = 63)	*P*-value
Steatosis grade 1	6 (33.3%)	7 (15.6%)	13 (20.6%)	0.016*
Steatosis grade 2	8 (44.4%)	10 (22.2%)	18 (28.6%)	
Steatosis grade 3	4 (22.2%)	28 (62.2%)	32 (50.8%)	
Lobular Inflammation grade 1	11 (61.1%)	21 (46.7%)	32 (50.8%)	0.529
Lobular Inflammation grade 2	7 (38.9%)	22 (48.9%)	29 (46.0%)	
Lobular Inflammation grade 3	0	2 (4.4%)	2 (3.2%)	
Portal Inflammation grade 0	5 (27.8%)	19 (42.2%)	24 (38.1%)	0.564
Portal Inflammation grade 1	13 (72.2%)	25 (55.6%)	38 (60.3%)	
Portal Inflammation grade 2	0	1 (2.2%)	1 (1.6%)	
Portal Inflammation grade 0	5 (27.8%)	19 (42.2%)	24 (38.1%)	0.286*
Portal Inflammation grade 1+2	13 (72.2%)	26 (57.8%)	39 (61.9%)	
Ballooning degeneration grade 1	10 (55.6%)	23 (51.1%)	33 (52.4%)	0.848
Ballooning degeneration grade 2	6 (33.3%)	18 (40.0%)	24 (38.1%)	
Ballooning degeneration grade 3	2 (11.1%)	4 (8.9%)	6 (9.5%)	
Fibrosis stage 0	2 (11.1%)	9 (20.0%)	11 (17.5%)	0.133
Fibrosis stage 1	10 (55.6%)	31 (68.9%)	41 (65.1%)	
Fibrosis stage 2	6 (33.3%)	5 (11.1%)	11 (17.5%)	

Non-obese means body mass index < 95th percentile and obese means body mass index ≥ 95th percentile.
P-value was calculated by Fisher's exact test. **P*-value was calculated by *Chi*-square test.
